# Environment Exploration and Colonization Behavior of the Pea Aphid Associated with the Expression of the *foraging* Gene

**DOI:** 10.1371/journal.pone.0065104

**Published:** 2013-05-29

**Authors:** Sophie Tarès, Laury Arthaud, Marcel Amichot, Alain Robichon

**Affiliations:** 1 INRA (Institut National de la Recherche Agronomique), UMR 1355 Institut Sophia Agrobiotech, Sophia-Antipolis, France; 2 CNRS (Centre National de la Recherche Scientifique), UMR 7254 Institut Sophia Agrobiotech, Sophia-Antipolis, France; 3 Université Nice Sophia Antipolis, UMR Institut Sophia Agrobiotech, Sophia-Antipolis, France; University of Arizona, United States of America

## Abstract

Aphids respond to specific environmental cues by producing alternative morphs, a phenomenon called polyphenism, but also by modulating their individual behavior even within the same morph. This complex plasticity allows a rapid adaptation of individuals to fluctuating environmental conditions, but the underlying genetic and molecular mechanisms remain largely unknown. The *foraging* gene is known to be associated with behavior in various species and has been shown to mediate the behavioral shift induced by environmental changes in some insects. In this study, we investigated the function of this gene in the clonal forms of the pea aphid *Acyrthosiphon pisum* by identifying and cloning cDNA variants, as well as analyzing their expression levels in developmental morphs and behavioral variants. Our results indicate that the expression of *foraging* changes at key steps of the aphid development. This gene is also highly expressed in sedentary wingless adult morphs reared under crowded conditions, probably just before they start walking and foraging. The cGMP-dependent protein kinase (PKG) enzyme activity measured in the behavioral variants correlates with the level of *foraging* expression. Altogether, our results suggest that *foraging* could act to promote the shift from a sedentary to an exploratory behavior, being thus involved in the behavioral plasticity of the pea aphid.

## Introduction

Aphids are insects which respond quickly to environmental changes by developing alternative phenotypes, such as asexual and sexual forms, a phenomenon called polyphenism. Asexual clonal forms produced during all spring and summer develop efficient strategies to adapt themselves to fluctuating conditions of their environment. Under conditions of reduced food quantity or quality, or when attacked by predators, clonal forms can switch in two generations from wingless to winged forms that easily colonize new host plants [1], [2]. In addition to the production of winged morphs, which are in charge of long range dispersion, clonal forms are able to react in a very short time using alternative escape behaviors for short range dispersion. Some aphids indeed leave their host plant and begin to explore their close environment to find fresh resources and to immediately settle new colonies [3], [4] The release of volatile compounds, such as the alarm pheromone secreted by aphids in the presence of predators, also triggers various reactions such as the withdrawal of the stylet from the plant, or the walking or dropping off the host plant [5]. This high behavioral plasticity makes them good candidates to explore the molecular basis of such a phenomenon. Nevertheless, almost nothing is known of the physiological and genetic mechanisms controlling polyphenism and individual behavior in aphids [6], [7].

A natural polymorphism in the *foraging* gene (*for*), that encodes a cGMP-dependent protein kinase (PKG), has been shown to be associated to behavioral plasticity in several insect species [8]. In the fruit fly *Drosophila melanogaster*, larvae and adults of the *rover* phenotype have larger moving trails in the presence of food compared to *sitters* [9], [10]. Higher levels of *for* expression and PKG activity are observed in the heads of the *rover* allelic variants [11]. The *for* gene was then suggested to play a role in the behavioral plasticity in response to food deprivation [12]. In the honeybee *Apis mellifera,* higher levels of expression of the *for* gene (*Amfor*) and PKG activities were detected in foragers compared to nurses [13]. A subsequent study evidenced an *Amfor* expression peak during the period of transition from nurse to forager, and suggested that in normal conditions the honeybee foraging behavior is at least partly due to a trigger-effect of *Amfor* [14]. The *Amfor* expression was thus strongly associated with tasks performed outside the hive and suggested to affect the division of labor by modulating phototaxis [15], [16]. A differential expression of the *for* gene homologues has also been reported in other social insects although a negative correlation was initially observed between the PKG activity level and foraging activities. In the common wasp *Vespula vulgaris*, bumblebees and the some red harvester ants in the genus *Pogonomyrmex*, which all display progression of worker tasks during their lifespan, known as age-related polyethism, lower *for* mRNA levels were detected in foraging workers than in nest workers [17]–[19]. Nevertheless, Ingram et al. [20] recently highlighted a more complex expression pattern of the *for* gene in the harvester ant foragers : *for* expression changes in the course of the day consistently with the task-specific circadian rhythm observed in this species. A similar fluctuation of the *for* expression level has been observed in honeybee over the time [14]. A more precise and systematic study of the *for* gene expression combined with a meticulous analysis of behavioral traits is likely the best approach to understand the mechanisms by which *for* modulates the insect behavior.

The recent sequencing of the pea aphid *Acyrthosiphon pisum* genome and its subsequent automated annotation [21] has provided an appreciable tool to characterize the *for* gene in a clonal species displaying high behavioral plasticity. In this study, we first cloned the cDNAs of the pea aphid *foraging* gene orthologue *Apfor*. We then analyzed their expression patterns across parthenogenetic developmental stages, morphs and behavioral variants. We also explored whether environmental conditions (low and high population densities, which determine food resources availability) may impact *Apfor* expression. Our experiments showed that *Apfor* is highly expressed in key developmental stages and in wingless adults reared under crowded conditions. The PKG enzyme activity measured in the behavioral variants corroborates the observed variations of *Apfor* expression. We then suggest that *Apfor* could possibly act to trigger the shift from sedentary to exploratory behavior. Our results lay the groundwork for a more detailed analysis of the implication of *Apfor* in the behavioral plasticity of the pea aphid.

## Materials and Methods

### Aphid strain

The YR2 clone of the pea aphid *Acyrthosiphon pisum* was provided by Denis Tagu (INRA, Rennes, France). It naturally harbors the secondary endosymbiont *Regiella insecticola*. Aphids were reared on 15 centimeters diameter flowerpot containing 3 broad bean *Vicia fabae* plants at 18°C under a 16/8 light/dark cycle that ensures parthenogenic reproduction (virginiparous females). Aphids were reared in parallel at low and high population densities.

### Cloning of the *A. pisum foraging* gene

PolyA+ mRNAs were extracted from 100 mg of whole homogenized *Acyrthosiphon pisum* wingless viviparous adult females using the Micro mRNA purification kit (GE Healthcare). Specific cDNAs were amplified using 0.5 µg of polyA+ mRNA according to the BD Smart^™^ RACE cDNA amplification kit followed by the BD Advantage™ 2 PCR Enzyme System protocol (BD Biosciences) with specific primers designed from an EST sequence available in the AphidBase (www.aphidbase.com) and encoding a partial *for* cDNA (sense primer : GAGTGGAGGTGAGCAGAG, antisense primer : CACTTTTCCGGAGGTCATAG). These primers match with a region of the first tandem cGMP-binding motif of the *for* gene. Amplified fragments were cloned with the TA Cloning® kit (Invitrogen) using the pCR®2.1 vector and transformed into electrocompetent One shot® TOP10 *E. coli* cells (Invitrogen). Recombinant plasmids from positive clones were extracted, purified and sequenced by GATC Biotech (Konstanz, Germany). The obtained sequences were tested for homology with known *for* genes using NCBI database. Two sequences homologous to *for* were then identified and named variant 1 and variant 2. Primers were designed from these two sequences to amplify the corresponding full length cDNAs from freshly extracted polyA+ mRNA samples (variant 1 mRNA sense primer : ACTGTTGCTTCAGTCGCTGTTTACA, variant 2 mRNA sense primer : CAGCGTCTATCTACGTATGTGC, common antisense primer : GTAAAATCGTTGAGGCGGACAA). The absence of *for* homologous genes in the YR2 endosymbionts was confirmed using public annotations of genomes of the primary symbiont *Buchnera aphidicola* and the secondary symbiont *Regiella insecticola.*

### Collection of behavioral variants

Behavioral variants were obtained under two different conditions of population density. Wingless viviparous adults (VWL) were recovered under low population density conditions that provide good food quality and large space for female reproduction. For that purpose, five females were transferred on a flowerpot and their one day-old adult progeny sucking on the leaves was collected at the same time in the morning. The other behavioral variants were obtained under high population density initiated from five females transferred on a flowerpot and left there giving rise to several generations until crowded conditions were achieved. Generated winged aphids (VW) were then collected when they were one day-old (low survival without fresh plant). In the same time, some wingless adult aphids left the plants, starting to walk and explore their environment (named wingless forager adults VWLf) while others kept feeding on stems or leaves (named wingless sedentary crowded adults VWLc). VWLf and VWLc were collected at the same time about 3 hours after the first individuals started walking on the cage walls, which could be whenever in the daytime.

### Quantitative real-time PCR assays

PolyA+ mRNAs were extracted as described above from 100 mg of whole *Acyrthosiphon pisum* individuals at various developmental stages : 1st instar larvae (L1) (about 300 insects), 2nd instar larvae (L2) (about 250 insects), winged (L3W) and wingless (L3WL) 3rd instar larvae (about 150 insects each), winged (L4W) and wingless (L4WL) 4th instar larvae (about 50 insects each), winged (VW) and wingless viviparous adult (VWL) (about 40 insects each). L1 to L4 were sampled 2 days after the molt and adults were collected at 1 day-old. Behavioral variants (about 40 insects of each VW, VWL, VWLc and VWLf) were sampled in conditions as described above. Experiments were performed on whole aphids and not on aphid heads because isolation of heads from L1 and L2 was difficult and time consuming.

cDNAs were transcribed using the SuperScript™II reverse transcriptase (Invitrogen) in accordance with the supplier's instructions using 1 µg of polyA+ mRNAs. The efficiency of each reverse transcription reaction was tested by amplifying the obtained cDNAs with the different primers sets used in the following qPCR reactions using the UptiTherm DNA polymerase (Uptima). The qPCR reactions were performed for each cDNA sample using the qPCR MasterMix Plus for SYBR® Green I No Rox (Eurogentec, Belgium) on a DNA Engine Opticon®2 instrument (Bio-Rad) with the following qPCR conditions : 50°C for 2 min, 95°C for 7 min, 40×(95°C for 30 sec, 60°C for 30 sec, 72°C for 45 sec) and specific primers (Table S1).

An internal fragment of the Rpl7 ribosomal protein of *A. pisum* (Genbank accession NM001135898) was used as a control to normalize the *for* expression (RPL7-2QPCR Forward: ACTGTTCAGATTGCGTCAGATC, RPL7 -2QPCR reverse: AGTTCCCTTACGCTCTTCAAGT). All reactions were carried out in triplicates for each cDNA prepared from at least four independent mRNA extractions. Quantification of relative mRNA levels was calculated using the ΔΔCt method [22]. The mean and standard error were calculated for each experimental condition. The values obtained for expression levels were relative and could only be compared within each experimental run. Statistical analyses were performed using a one-way ANOVA followed by a Fisher's PLSD (Protected Least Significant Difference) to test for significant differences of expression between developmental stages or behavioral variants.

### PKG enzyme activity assays

PKG enzyme activity assays were performed using the Cyclex cyclic GMP dependent protein kinase (cGK) assay kit from Cyclex Co, Ltd. Fresh whole bodies and cut heads from the different behavioral variants were separately homogenized on ice in the provided kinase buffer. Samples were centrifuged for 5 min and supernatants were quantified for total protein amount using the Coo protein assay reagent (Uptima) by the Bradford method. 5 µg of total proteins were then analyzed for PKG enzyme activity, with the following controls to ensure the PKG specificity of the reactions : blank (complete reaction buffer), positive control (complete reaction buffer with a cGK positive control) and negative controls (samples in reaction buffer without cGMP or without ATP and cGMP, samples in complete reaction buffer added with the protein kinase inhibitor K-252a from Sigma). OD was quantified at dual wavelengths of 450/540 nm in a Spectramax Plus 384 spectrophotometer (Molecular Devices). The PKG enzyme activity was expressed as the OD for 5 µg of total proteins. The mean and standard error were calculated for each experimental condition. Statistical analyses were performed using a one-way ANOVA followed by a Fisher's PLSD to test for significant differences between behavioral variants.

## Results

### The Acyrthosiphon pisum foraging gene

**Figure 1:**
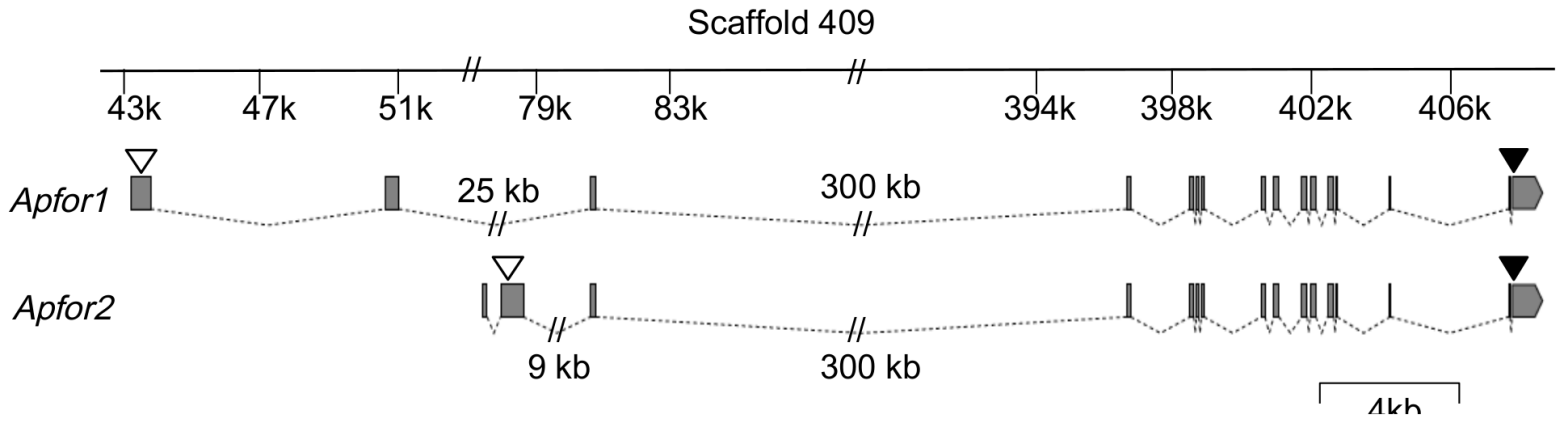
Structure of the *Apfor1* and *Apfor2* transcripts. Exons 3–16 are identical, only exons 1 and 2 differs as the result of alternative splicing. Scaffold 409 containing the two transcripts is represented at the top. Exons are indicated by grey boxes and introns by dotted lines. White triangles indicate the position of start codons, black triangles indicate the position of the stop codon.

To isolate *A. pisum for* cDNAs, we designed primers from an *A. pisum* EST fragment available on the Aphidbase 1.0 database that showed unambiguous homology with the *Drosophila melanogaster for* gene. 3' and 5' RACE experiments resulted in the cloning of two full-length cDNA sequences we named *Apfor1* (GeneBank accession number JN812212) and *Apfor2* (GeneBank accession number JN812213). Thanks to the recent sequencing of the pea aphid genome, we managed to locate these two complete *A. pisum for* cDNAs on a genomic scaffold (Scaffold409, GeneBank accession number GL350029) and subsequently deduced that they are composed of 16 exons, exons 3 to 16 being common to *Apfor1* and *Apfor2* (Figure 1). The full size of the *Apfor* genomic sequence is difficult to determine because of the large size of some introns (notably intron 3 covering more than 300 kb) and of residual sequencing errors. In agreement with genome sequence data, a Southern blot analysis clearly showed that only one gene homologous to *for* is present in the *A. pisum* genome (data not shown).

Figure S1 shows the nucleotide and deduced amino acid sequences of the two complete cDNAs. These sequences are 3420 and 3338 bp long respectively, and contain an ORF of 2331 bp for *Apfor1* and 2112 pb for *Apfor2*. They differ only in their 5' region (the first two exons) and perfectly overlap for their following 2462 bp (Figure 1), as a result of alternative splicing. Exons 1 and 2 of *Apfor2* are indeed located in the intron 2 of the *Apfor* gene suggesting that these exons are spliced in the *Apfor1* variant. Both *Apfor1* and *Apfor2* sequences are about 56% identical at the nucleotide level and 70% similar at the amino acid level to the *for* gene sequence of *D. melanogaster*. Analysis of the two deduced amino acid sequences reveals typical structures of a cGMP-dependent protein kinase including a N-terminal region containing a regulatory compartment (a dimerization domain with a leucine zipper motif, autophosphorylation sites and an autoinhibitory domain), two tandem cyclic nucleotide-binding domains and a serine/threonine kinase catalytic domain as determined by PROSITE at SIB ExPASy Bioinformatics Resource Portal (http://prosite.expasy.org). Despite their differences in the 5' region, both transcripts encode putatively complete and active PKG proteins suggesting they are two functional alternative splicing variants of the same gene.

### Comparative expression of *Apfor* in morphs and developmental stages

**Figure 2:**
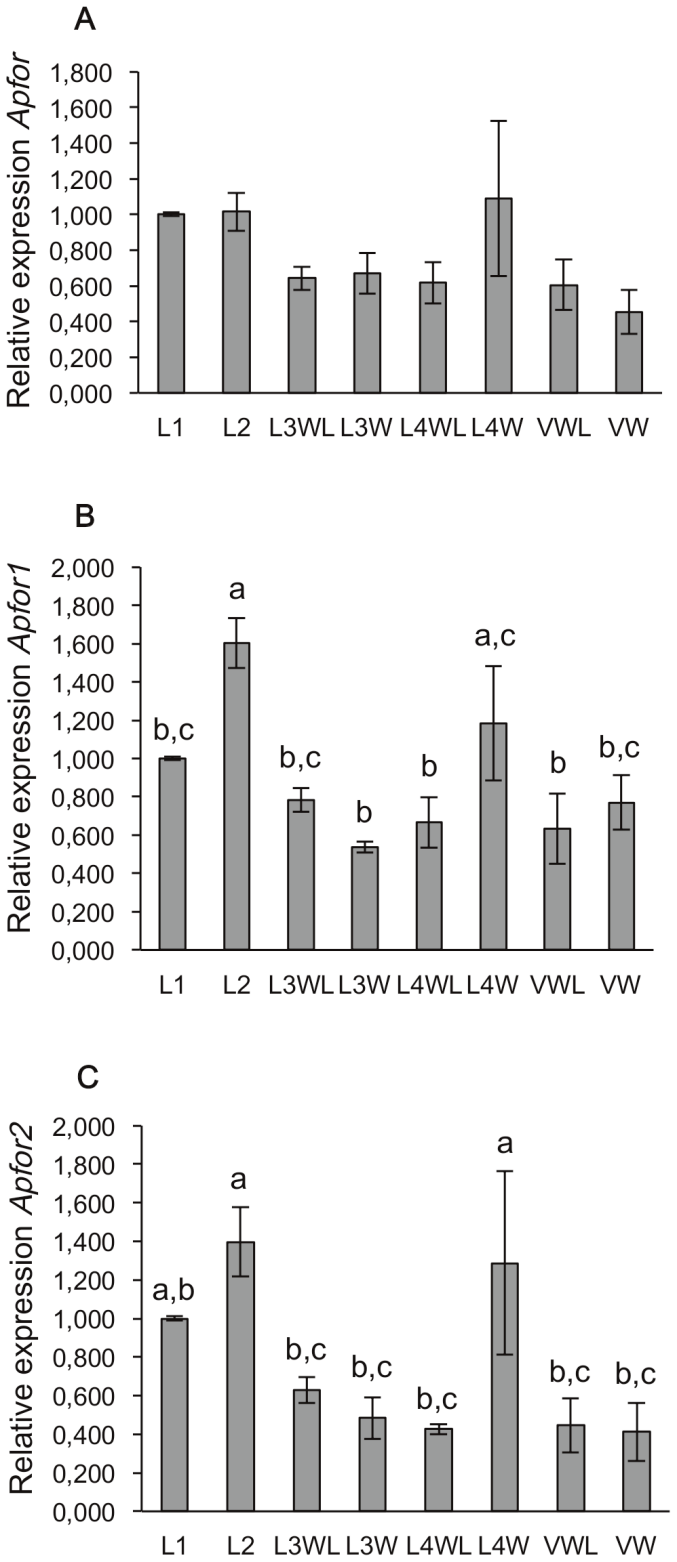
Relative expression of *Apfor*, *Apfor1* and *Apfor2* in the different developmental stages of pea aphids. Quantitative RT-PCRs were done using whole bodies from winged and wingless developmental stages of larvae and adults. (A), Global relative expression of *Apfor*. (B), Relative expression of *Apfor1*. (C), Relative expression of *Apfor2*. LI, 1st instar larvae; L2, 2nd instar larvae; L3WL, wingless 3rd instar larvae; L3W winged 3rd instar larvae; L4WL, 4th instar larvae; L4W, winged 4th instar larvae; VWL, wingless viviparous adults, VW, winged viviparous adults. Relative expression is normalized to L1 stage. Errors bars represent the standard errors converted to the same arbitrary scale as the means. A one-way ANOVA followed by a Fisher's PLSD test shows the statistically significant differences between groups denoted by different letters (*P*<0,05 or *P*<0,01).

The relative abundance of *Apfor* transcripts was determined among winged (obtained under high population density) and wingless morphs (reared under low population density) of the different developmental stages (L1 to adult) using quantitative real-time PCR assays. A first set of primers, designed to potentially amplify all the different *Apfor* transcripts (Table S1), targeted a conserved region overlapping the exons 15 and 16 at the 3' end of the kinase domain. Results indicated that *Apfor* is similarly expressed in winged and wingless morphs during development (one-way ANOVA followed by Fisher's PLSD tests, F = 1,67, *P*>0,05) (Figure 2A). Nevertheless, a noticeable increase of *Apfor* expression could be detected for the L1, L2 and L4W stages.

Two other sets of primers, targeting the second exon of each transcript, were designed to specifically amplify each of the two *Apfor* transcripts (Table S1). The expression of *Apfor1* is significantly higher in the L2 and L4W stages than in other stages (F = 5,12; *P*<0,001 and *P*<0,05 respectively) (Figure 2B). A similar difference in the expression levels is observed for *Apfor2* (F = 4,06; *P*<0,05 for both L2 and L4W stages) (Figure 2C). The expression patterns of the two transcripts are thus roughly similar during pea aphid development.

Northern blot experiments using a fragment overlapping the end of the first and half of the second cGMP-binding domains as a specific probe, indicated the presence of at least two additional *Apfor* transcripts (Figure S2) we failed to clone probably because of their weak expression.

### Comparative expression of *Apfor* among behavioral variants of pea aphid adults

**Figure 3:**
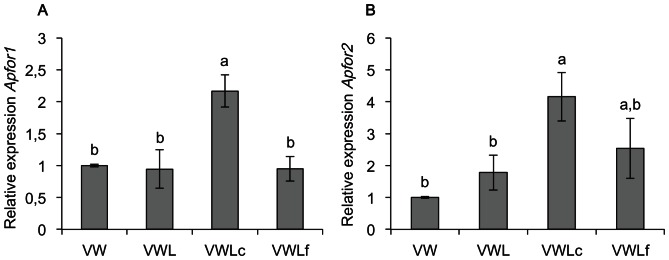
Relative expression of *Apfor1* and *Apfor2* among behavioral variants of adults pea aphids. (A), Relative expression of *Apfor1*. (B), Relative expression of *Apfor2*. VW, winged viviparous adults reared under high population density; VWL, wingless viviparous adults reared under low population density; VWLc, wingless viviparous adults reared under high population density; VWLf, wingless viviparous adults foragers reared under high population density. Errors bars represent the standard errors converted to the same arbitrary scale as the means. Relative expression is normalized to VW. A one-way ANOVA followed by a Fisher's PLSD test was performed. The statistically significant differences between groups are denoted by different letters (*P*<0,01 in case of *Apfor1* and *P*<0,05 in case of *Apfor2*).

Under crowded conditions, different behavioral variants are observed in wingless adults, in addition to the typical production of winged morphs able to disseminate over a long distance [1], [5]. Indeed, some wingless aphids keep on feeding on the phloem sap under the leaves or on the stems (variants VWLc) while others leave the plant, walk and forage their environment to find better conditions for feeding and producing offspring (variants VWLf). We thus analyzed the level of both *Apfor* transcripts in four categories of behavioral adult variants, three reared under high population density (winged adults VW, wingless sedentary crowded adults VWLc and wingless forager adults VWLf) and one reared under low population density (wingless sedentary adults VWL) (Figure 3). The expression of the *Apfor1* transcript is significantly higher in the VWLc variants compared to other variants (one-way ANOVA followed by Fisher's PLSD test; F = 8,71, *P*<0,001) (Figure 3A). *Apfor2* transcripts are also significantly more expressed in the VWLc variant (F = 4,08, *P*<0,05) but we observe the same trend in the VWLf variant. A higher standard error value is observed for both variants (Figure 3B). These results confirm our previous observations (Figure 2) that no significant difference occurs in *Apfor* expression between VW reared under high population density and VWL reared under low population density. So, the high expression level of the pea aphid *for* gene seems to be associated with the exploratory behavior due to crowded conditions rather than with morphological variants.

### PKG enzyme activity among behavioral variants of pea aphid adults

**Figure 4:**
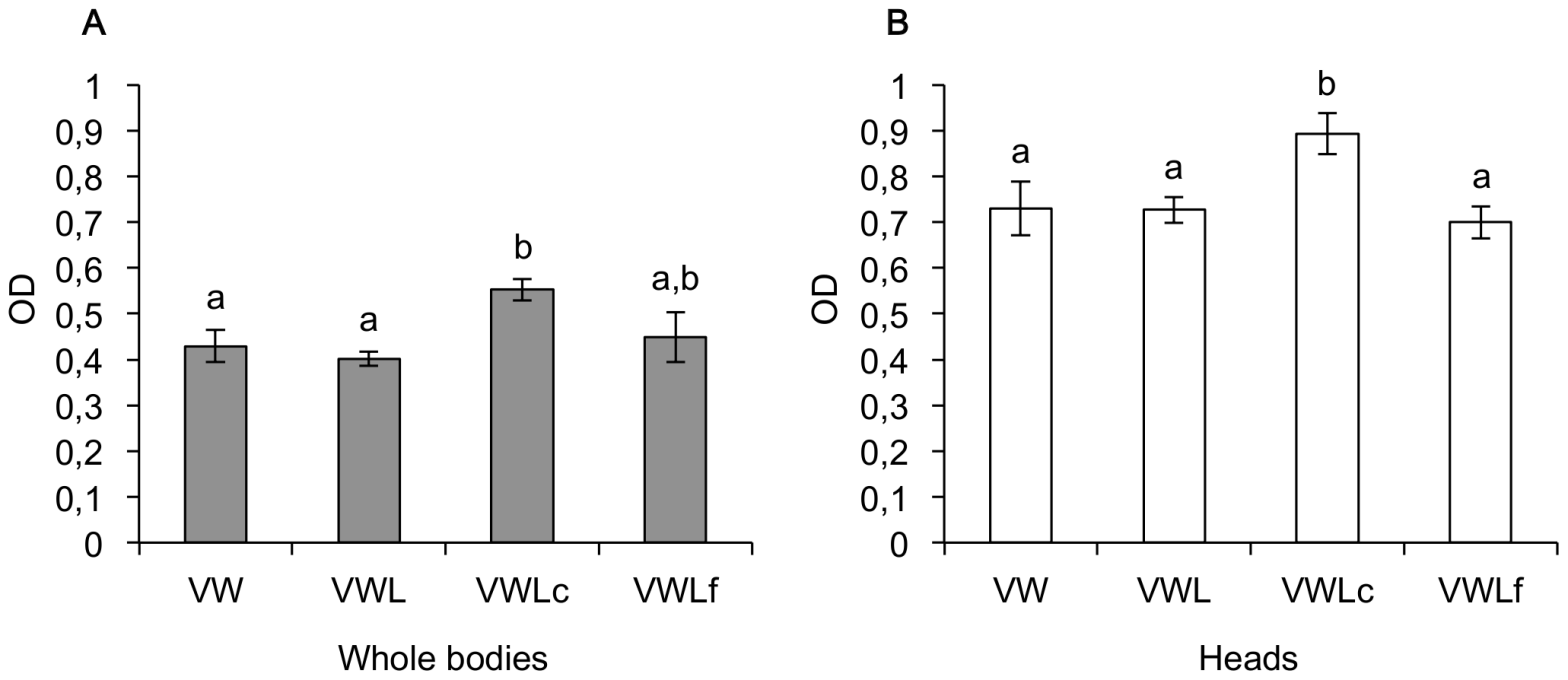
PKG enzyme activity among behavioral variants of adults pea aphids. (A) PKG enzyme activity in whole bodies. (B) PKG enzyme activity in heads. PKG enzyme activity is expressed as the OD for 5 µg of total proteins for each behavioral variant. Error bars represent the standard errors converted to the same arbitrary scale as the means. A one-way ANOVA followed by a Fisher's PLSD test was performed. The statistically significant differences between groups denoted by different letters (*P*<0,05).

PKG enzyme activities, that represent at least the combined activities of the two *Apfor* variants, were comparatively measured in the different behavioral variants from whole bodies or heads. Figure 4 shows PKG enzyme activity in whole bodies and in heads of all behavioral variants, confirming that *Apfor* transcripts produce functional PKG proteins. PKG enzyme activity pattern is roughly similar using whole bodies or heads, indicating that measurements on whole bodies are certainly representative of what happens in heads. PKG enzyme activity pattern evidenced in heads among behavioral variants correlates with the *Apfor* expression pattern (Figure 3) and thus with the probable implication of the PKG in the aphid behavior. Indeed, the VWLc variant displays a significantly higher PKG enzyme activity in whole bodies as in heads (F = 3,116, *P*<0,05).

## Discussion

Our results confirm that *foraging*, an important gene associated with behavioral plasticity in insects, is conserved in Hemiptera. We indeed report the cloning and analysis of the transcripts of this gene in the pea aphid *Acyrthosiphon pisum*, a particularly interesting species regarding its polyphenism ability combined with behavioral plasticity, allowing rapid adaptation to unfavorable environmental conditions, such as overcrowding or the presence of enemies. This short-term adaptive response confers to aphid species their remarkable invasive potential which make them efficient insects pests.

Interestingly, the expression of the pea aphid *foraging* gene seems as complex as reported in *D. melanogaster* [23] or *Cænorhabditis elegans* [24] with multiple alternative splicing transcripts. The functional significance of the two identified transcripts was not determined but they are full length variants which encode two putative functional proteins. These transcripts are produced by a single gene as determined by a Southern blot experiment (data not shown). The analysis of sequences from the pea aphid genome reveals a second gene coding for a cGMP-dependent protein kinase (GeneBank, accession number XM 001947008), very likely the ortholog to the *dg1* gene from *D. melanogaster*. These two aphid genes have diverged enough (41% of similarity) not to cross-hybridize using classical Southern blot techniques. The pea aphid seems thus as well genetically equipped as *D. melanogaster* or honeybee to set up behavioral plasticity.

In a first step, we tested whether the morphological state (wingless or winged morphs) and the different developmental stages of the viviparous parthenogenetic pea aphids could be associated with a differential *Apfor* expression. The expression patterns of *Apfor1* and *Apfor2* transcripts are roughly similar, and no significant difference is found between wingless and winged morphs at any developmental stage. By contrast, we observe that the 2nd instar and the winged 4th instar larval stages show a significantly higher expression of the two *Apfor* transcripts than the other stages. The L2 stage has previously been shown to be crucial for wings formation. Indeed, Ishikawa and colleagues [25] demonstrated that all first instar larvae (reared under low or high density conditions) possess wing primordia which degenerate during the 2nd instar larvae in the wingless forms only. In the winged forms, the wing primordia develop and become thick. In the same way, these authors showed that during the 4th instar larval stage the transition of internal structures in wing buds is dramatic: the muscle cells completely proliferate and fuse into syncitial muscle cells. *Apfor* is thus highly expressed at key steps of the larval development involved in wing formation and thus in the flight capacity of the pea aphid.

In a second step, we tested the expression of *Apfor* among the behavioral variants of viviparous parthenogenetic adults which are produced under low population density or crowded environmental conditions. Surprisingly, behavioral variants having a significantly higher *Apfor* expression are wingless aphids feeding on phloem sap from leaves or stems. The foragers, which escape to find fresh resources, present only a slight increase of *Apfor2* transcripts. As our results were obtained using whole aphid body and not only head, which is the control center of the behavior, a direct correlation between *Apfor* and the aphid behavior could not be inferred. Indeed, the *for* gene has also been shown in Drosophila to be implicated in other physiological processes such as cristal cells formations [26] or modulation of the cardiac rythm in Drosophila [27]. We thus performed measurements of the PKG enzyme activity in whole bodies and in heads of the different behavioral variants. The results parallel those observed for the *Apfor* expression, with a significant higher activity in VWLc aphids. This reinforces the hypothesis of an existing correlation between *Apfor* expression and aphid behavior. Taken together, our results suggest that *Apfor* may induce a foraging behavior in some wingless adults under crowded conditions acting as a promoting signal to find better environmental niches in a short delay. Foragers would have a weaker *Apfor* transcript and PKG activity level, maybe as a residual expression after the behavioral switch initiated by *Apfor*. Considering that a stimulus is certainly required to shift from a sedentary to a dispersal behavior, the *for* gene is a promising candidate to fulfill this role. Complementary behavioral experiments are now needed to determine unambiguously whether wingless sedentary adults reared under crowded conditions and presenting an increased *Apfor* level are those which subsequently forage their environment. To our knowledge, the detailed behavior of wingless adult aphids has not been described yet. In addition, the exact nature of the stimulus and its molecular targets still have to be identified.

In the honeybee *A. mellifera*, the occurrence of a peak in *Amfor* expression, rather than a slowly upcoming and continuous high expression level, suggests that the transition from nurses to foragers outside the hive is triggered by *Amfor* [14]. In the same way, in the harvester ant *P. occidentalis* a daily fluctuation occurs in the expression of the *for* ortholog *Pofor*. A peak of mRNA level is observed in foragers at midday [19] while nest workers show lower levels of *Pofor* mRNA during the day and similar or higher levels in late evening and early morning hours. Interestingly, in a related harvester ant *P. barbatus*, Ingram and colleagues [17] showed a lower expression of *Pbfor* in foragers than in workers but the study was limited to the collection of workers at a single time in the day (as we did with pea aphids). As the sampled workers were collected in the field in early morning, it cannot be excluded that the *Pbfor* expression fluctuates in a similar pattern as *Pofor* in the daytime. In laboratory conditions, the population density of pea aphids had to be very high with confluent adults to simultaneously allow the production of winged morphs and wingless behavioral variants. As a consequence, it was very difficult to collect synchronous individuals for all qPCR replicates, which might thus explain the high range of standard errors for our experiments (Figure 3). As in the harvester ants, the *Apfor* gene might be highly expressed at a specific time in the day or in the life cycle of only part of pea aphid wingless individuals, in response to an external stimulus triggering their foraging behavior. Complementary experiments are needed to determine the timing of *Apfor* expression in wingless pea aphids under crowded conditions, and to question whether all individuals or only some of them differentially express *Apfor* in order to establish a specific relationship between this gene and behavior plasticity. As in other species, the nature of the interacting stimuli and their molecular targets remain also to be determined. In nematodes, odors and pheromones act to stimulate and regulate the expression of the *for* ortholog *Egl-4* [29] and in the nematode *Pristionchus pacificus*, *Ppa-egl-4* is directly implicated in the attraction to the pheromone emitted by its insect host [30]. Could the pea aphid alarm pheromone, which mediates the production of winged dispersal morphs, also regulate *Apfor* expression? In this case, a new role in chemo-attraction or olfaction could be assigned to *for* in insects alike to its role in nematodes.

As in social insects whose task behaviors appear to be associated with the regulation of the *for* gene expression, the fluctuations of *Apfor* expression in pea aphids seems to be associated with feeding-behavior plasticity. This would establish a link between the *for* gene and the plasticity of the feeding behavior across the insect class. In parthenogenetic insects like aphids, among which some species reproduce only as clonal forms, the existence of such genes contributing to adaptation to environmental stresses is very important to compensate for the lack of the genetic variability produced by mating. These genes may allow aphids to reduce the delay in response to harmful biotic (poor quality of food resources, presence of natural enemies) and abiotic factors (pollutants, climate) and to develop rapid adaptive responses to environmental cues by producing the best adapted phenotypes. Finally, the universality of *foraging* as a molecular modulator of behavior seems to be strengthened.

## Supporting Information

Figure S1 **Nucleotide sequences, deduced protein sequences and structure of the two cDNA variants of** ***Apfor*** **.** The two variants are noted v1 (*Apfor1*) and v2 (*Apfor2*). The characteristic amino acid signature of the leucine zipper motif inside the dimerization domain is boxed in yellow. The key motif of the autoinhibition domain is boxed in green. Exon limits are indicated with vertical blue bars. Nucleotides and amino acids are numbered on the right.
(PDF)

Figure S2 **Northern blot analysis of the** ***Apfor*** **expression.** 6 µg of polyA+ mRNAs from wingless adults were used. The 406 bp probe overlapping the two cGMP-binding domains of *Apfor* was digoxigenine-labelled using the PCR DIG probe synthesis kit from Roche Diagnostics (Germany). A RPL7 fragment was used as control.
(PDF)

Table S1 **Oligonucleotide primers used for quantitative real-time PCR.**
(PDF)

## References

[1] Sutherland ORW (1969) The role of crowding in the production of wing forms by two strains of the pea aphid *Acyrthosiphon pisum* . J Insect Physiol 15: 1385–1410.

[2] Müller CB, Williams IS, Hardie J (2001) The role of nutrition, crowding and interspecific interactions in the development of winged aphids. Ecol Entomol 26: 330–340.

[3] Tabadkani SM, Ahsaei SM, Hosseininaveh V, Nozari J (2012) Walking faster to restore energy loss! Food stress drives a shift between reproductive and dispersal phases in apterous pea aphids. Physiol Behav. In press. doi:10.1016/j.physbeh.2012.12.004 10.1016/j.physbeh.2012.12.004 23262143

[4] Dill L M, Fraser AHG, Roitberg BD (1990) The economics escape behavior in the pea aphid, *Acyrthosiphon pisum* . Oecologia 83: 473–478. 28313180 10.1007/BF00317197

[5] Montgomery ME, Nault LR (1977) Comparative response of the aphids to the alarm pheromone, (E)-beta-farnesene. Ent Exp Appl 22: 236–242.

[6] Huybrechts J, Bonhomme J, Minoli S, Prunier-Leterme N, Dombrovsky A, et al (2010) Neuropeptide and neurohormone precursors aphid, *Acyrthosiphon pisum* . Insect Mol Biol 19: 87–95. 10.1111/j.1365-2583.2009.00951.x 20482642

[7] Simon JC, Pfrender EM, Tollrian R, Tagu D, Colbourne KJ (2011) Genomics of Environmentally Induced Phenotypes in 2 Extremely Plastic Arthropods. J Hered 102: 512–525. 21525179 10.1093/jhered/esr020 PMC3156564

[8] Reaume CJ, Sokolowski MB (2011) Conservation of gene function in behaviour. Phil Trans R Soc 366: 2100–2110. 10.1098/rstb.2011.0028 PMC3130371 21690128

[9] Sokolowski MB (1980) Foraging strategies of *Drosophila melanogaster* : a chromosomal analysis. Behav Genet 10: 291–309. 6783027 10.1007/BF01067774

[10] Pereira HS, Sokolowski MB (1993) Mutations in the larval foraging gene affect adult locomotory behavior after feeding in drosophila melanogaster. Proc Natl Acad Sci USA 90: 5044–5046. 8506349 10.1073/pnas.90.11.5044 PMC46650

[11] Osborne K, Robichon A, Burgess E, Butland S, Shaw RA, et al (1997) Natural behavior polymorphism due to a cGMP-dependent protein kinase of *Drosophila* . Science 277: 834–836. 9242616 10.1126/science.277.5327.834

[12] Kaun KR, Riedj CAL, Chakaborty-Chatterjee M, Belay AT, Douglas SJ, et al (2007) Natural variation in food acquisition mediated via a cGMP-dependent protein kinase. J Exp Biol 210: 3547–3558. 17921156 10.1242/jeb.006924

[13] Ben-Shahar Y, Robichon A, Sokolowski MB, Robinson GE (2002) Influence of gene action across different time scales on behavior. Science 296: 741–744. 11976457 10.1126/science.1069911

[14] Heylen K, Gobin B, Billen J, Hu TT, Arckens L, et al (2008) *Amfor* expression in the honeybee brain : a trigger mechanism for nurse-forager transition. J Insect Physiol 54: 1400–1403. 18725227 10.1016/j.jinsphys.2008.07.015

[15] Ben-Shahar Y, Leung HT, Pak WL, Sokolowski MB, Robinson GE (2003) cGMP- dependent changes in phototaxis : a possible role for the foraging gene in honey bee division of labor. J Exp Biol 206: 2507–2515. 12796464 10.1242/jeb.00442

[16] Ben-Shahar Y (2005) The *foraging* gene, behavioral plasticity, and honeybee division of labor. C Comp Physiol A 191: 987–994. 10.1007/s00359-005-0025-1 16133503

[17] Ingram KK, Oefner P, Gordon DM (2005) Task-specific expression of the *foraging* gene in harvester ants. Mol Ecol 14: 813–818. 15723672 10.1111/j.1365-294X.2005.02450.x

[18] Lucas C, Sokolowski MB (2009) Molecular basis for changes in behavioral state in ant social behaviors. Proc Natl Acad Sci USA 106: 6351–6356. 19332792 10.1073/pnas.0809463106 PMC2669355

[19] Tobback J, Mommaerts V, Vandermissen HP, Smagghe G, Huybrechts R (2011) Age- and task-dependent *foraging* gene expression in the bumblebee *Bombus terrestris* . Arch Insect Biochem Physiol 76: 30–42. 21136525 10.1002/arch.20401

[20] Ingram KK, Kleeman L, Peteru S (2011) Differential regulation of the *foraging* gene associated with task behaviors in harvester ants. BMC Ecol 11: 19. 21831307 10.1186/1472-6785-11-19 PMC3180247

[21] The International Aphid Genomics Consortium (2010) Genome sequence of the pea aphid *Acyrthosiphon pisum* . PloS Biol 8: e1000313 doi:10.1371/journal.-pbio.1000313 20186266 10.1371/journal.pbio.1000313 PMC2826372

[22] Livak KJ, Schmittgen TD (2001) Analysis of relative gene expression data using real-time quantitative PCR and the 2(-Delta Delta C(T)) method. Methods 25: 402–408. 11846609 10.1006/meth.2001.1262

[23] Kalderon D, Rubin GM (1989) cGMP-dependent protein kinase genes in *Drosophila* . J Biol Biochem 264: 10738–10748. 2732245

[24] Fujiwara M, Sengupta P, McIntire SL (2002) Regulation of body size and behavioral state of *C. elegans* by sensory perception and the EGL-4 cGMP-dependent protein kinase. Neuron 36: 1091–1110. 12495624 10.1016/s0896-6273(02)01093-0

[25] Ishikawa A, Hingo S, Miura T (2008) Morphological and histological examination of polyphenic wing formation in the pea aphid *Acyrthosiphon pisum* (Hemiptera, Hexapoda). Zoomorphology 127: 121–133.

[26] Milchanowski AB, Henkenius AL, Narayanan M, Hartenstein V, Banerjee U (2004) Identification and characterization of genes involved in embryonic cristal cell formation during Drosophila hematopoeisis. Genetics 168: 325–339. 15454546 10.1534/genetics.104.028639 PMC1448098

[27] Johnson E, Sherry T, Ringo J, Dowse H (2002) Modulation of the cardiac pacemaker of Drosophila: cellular mechanisms. J Comp Physiol B 172: 227–236. 11919704 10.1007/s00360-001-0246-8

[28] Braendle C, Davis GK, Brisson JA, Stern DL (2006) Wing dimorphism in aphids. Heredity 97: 192–199. 16823401 10.1038/sj.hdy.6800863

[29] L'Etoile ND, Coburn CN, Eastham J, Kistler A, Gallegos G, et al (2002) The cyclic GMP-dependent protein kinase EGL-4 regulates olfactory adaptation in *C. elegans* . Neuron 36: 1079–1089. 12495623 10.1016/s0896-6273(02)01066-8

[30] Hong RL, Witte H, Sommer RJ (2008) Natural variation in *Pristionchus pacificus* insect pheromone attraction involves the protein kinase EGL-4. Proc Natl Acad Sci USA 105: 7779–7784. 18509055 10.1073/pnas.0708406105 PMC2409422

