# Supplementary material for: Environment Exploration and Colonization Behavior of the Pea Aphid Associated with the Expression of the foraging Gene
Source: PLoS One. 2013 May 29;8(5):e65104. doi: 10.1371/journal.pone.0065104 (PMC3667181; doi:10.1371/journal.pone.0065104)
Supplement: Figure S1 — Nucleotide sequences, deduced protein sequences and structure of the two cDNA variants of Apfor . The two variants are noted v1 (Apfor1) and v2 (Apfor2). The characteristic amino acid signature of the leucine zipper motif inside the dimerization domain is boxed in yellow. The key motif of the autoinhibition domain is boxed in green. Exon limits are indicated with vertical blue bars. Nucleotides and amino acids are numbered on the right. (PDF) [file pone.0065104.s001.pdf]

```
V1 GACCGGTACCGCATGTAGTGGTCGTCGTCGTTGTTCCGCGTTTAACTGTTGCTTCAGTCGCTGTTTACACCGTGGCACAC 80
V2                                                           TTTCCAGCGTCTATCTACGTAT 22

V1 GTGTTATTACGACCGATAATATACGCGTGTAGAATAAGAAGTCGTATTATTATTAATAATAACATTAATTTTATATGAAA 160
V2 GTGCGGTGTCCAACCGGTGTCCGTCAGCCGTCGCGTCATCAGTATACTTTTTTAGCAATAATATTAGTATATAGTATAAT 102

V1 TTTAAACGCCACCGTCTCTCCCAGTCGTCTCCGACAGCAATCGTTAAGTCCGGGGAGTGGGAAGGTTTTCGTTCCAATAA 240
V2 ATTCCGAACAAATAATAATTCGTTCCACTCGAGTTCCCTCAGCGTCGTTTGCGGTTCCGTCCTCGCAGCGTACGTACCTC 182

V1 GTTCCGTCTCGCCACCGCGGCGATCGTACCGCAACCGTAGGTCATTCGGTCGTCGTTTTCCCGCTATCACGGTCACGTCC 320
V2 CTAACGCGCGCGATCGGTGCATGTGTTTGTGTAATGCTCATAGATCCTTGACCCGGTTGTGTTGCTGCGGCAGTCTGTTC 262

                                          M  R  L  C  F  G  Q  W  C  I  P  W  P  G
V1 GCGAGGCATGCTCGCCCGCGAACAACAGTGGCTGCCTTTATGAGACTGTGTTTCGGCCAGTGGTGTATCCCGTGGCCTGG 400
V2 GCAACGAGACAGCGGAGGGGAAATCACATATTATACGCACGGAAAGCGTAAACGAAACAGCGCACCACCGCGCGTGTACG 342

    R  N  R  T  T  T  I  T  G  V  P  T  T  D  A  L  P  L  P  E  D  Y  D  Y  D  N  D
V1 CCGAAATAGAACGACCACGATTACCGGCGTGCCGACGACCGACGCATTGCCTCTGCCGGAAGACTACGACTACGACAACG 480
V2 ACAGTCGGTTCGACGCGTCCGAAACCACACTGCAGCTACATAACATAATAGTACGCGCCGCGATATACGCGTAAACGACG 422

     S  V  S  S  A  V  D  V  L  Y  A  A  E  E  P  M  T  R  A  L  Q  P  S  D  S  S
V1 ACAGTGTATCGTCGGCAGTAGACGTGTTGTACGCCGCCGAAGAACCGATGACGCGAGCGCTGCAGCCGTCCGACTCGTCG 560
V2 GTACTATAATTAGAGTTATCGCATAGTTTGTGGCGACCGAGCCGCGTGTTACACTCGCGATGAGCACGTCCGTGATGAAC 502
                                                              M  S  T  S  V  M  N

    S  S  A  D  M  A  A  S  V  D  A  Q  D  L  K  T  L  L  E  A  K  D  T  L  I  R  N
V1 TCGTCCGCAGACATGGCGGCTTCAGTAGATGCTCAAGATTTGAAAACCTTACTGGAAGCAAAGGATACTTTGATTCGTAA 640
V2 AGGTTACGGGACCTGGAGCGCGAGCTGGCCCTCCGGACGTCCGAGTCG------AGGAAGAAAGACGACTACATAGCCCG 576
    R  L  R  D  L  E  R  E  L  A  L  R  T  S  E  S        R  K  K  D  D  Y  I  A  R
                         Dimerization domain (Leucine zipper motif)
    L  E  T  L  L  Q  A  R  N  N  E  I  Q  E  L  R  S  H  L  D  M  F  Q  S  V  F  P
V1 CCTGGAAACGTTACTTCAGGCCAGGAACAACGAAATCCAAGAGCTTCGGTCTCATTTAGACATGTTTCAAAGTGTGTTTC 720
V2 TCTGGAGCACCGGCTCGACGAGCGTGACGCTAGTGTCCGCCACCTGCGCAACGAGATCGACAAGTTCCGACAGGTGGTTC 656
    L  E  H  R  L  D  E  R  D  A  S  V  R  H  L  R  N  E  I  D  K  F  R  Q  V  V  R

    F  S  N  P  V  S  P  T  S  T  L  H  H  P  H  H  V  V  A  A  P  V  H  Q  Q  Q
V1 CGTTTAGCAATCCAGTGTCACCCACTAGTACGCTGCATCATCCTCACCACGTGGTGGCCGCTCCCGTTCATCAACAACAA 800
V2 GGCCGCTCACCCATCACATTATGGCCATTCAGATGTCGGCCGACGATCTGGTCGTGTATGGTGGCGGCGGTGGCGGTGGT 736
    P  L  T  H  H  I  M  A  I  Q  M  S  A  D  D  L  V  V  Y  G  G  G  G  G  G  G
                                                                Autoinhibition domain
    S  Q  N  L  V  A  K  S  L  T  S  L  L  D  G  I  A  R  P  R  K  Q  R  A  Q  G  I
V1 AGCCAAAATCTCGTTGCTAAATCTCTTACGTCGCTTCTGGATGGTATAGCCAGGCCACGTAAACAGAGGGCGCAAGGTAT 880
V2 GGTTGCTGTAACGGCGGCGATCATCACGGCCACGGCTCGGGCTCCTTGTTCAAGGGCCGGCCCACTCGG---CAGGCCAT 813
    G  C  C  N  G  G  D  H  H  G  H  G  S  G  S  L  F  K  G  R  P  T  R     Q  A  I

    S  A  E  P  Q  S  L  S  T  I  Q  E  L  S  Q  K  K  F  P  T  Y  P  K  N  D  R
V1 ATCTGCGGAGCCCCAGAGCCTAAGTACCATACAAGAACTGAGCCAAAAGAAATTCCCTACTTATCCGAAAAACGACAGG 960
V2 TTCGGCCGAACCG------CTCAGGACCACTGACCCGCTGCCTATCGTCAAGGTTCCA---------AAGTCTTCAAAG 878
    S  A  E  P        L  R  T  T  D  P  L  P  I  V  K  V  P           K  S  S  K

    S  R  E  L  I  K  G  A  I  L  D  N  D  F  M  K  N  L  E  S  T  Q  I  R  E  I  V
V1 TCTAGGGAATTGATCAAAGGTGCTATTTTGGACAATGACTTCATGAAAAACTTGGAATCCACACAGATTAGGGAAATAGT 1040
V2 TCTAGGGAATTGATCAAAGGTGCTATTTTGGACAATGACTTCATGAAAAACTTGGAATCCACACAGATTAGGGAAATAGT 958
    S  R  E  L  I  K  G  A  I  L  D  N  D  F  M  K  N  L  E  S  T  Q  I  R  E  I  V

     D  C  M  Y  P  V  E  Y  A  S  D  S  I  I  I  K  E  G  D  V  G  S  I  V  Y  V  M
V1 GGACTGTATGTATCCCGTTGAGTATGCCTCCGACAGCATTATTATTAAAGAAGGAGATGTGGGCAGTATTGTTTATGTTA 1120
V2 GGACTGTATGTATCCCGTTGAGTATGCCTCCGACAGCATTATTATTAAAGAAGGAGATGTGGGCAGTATTGTTTATGTTA 1038
     D  C  M  Y  P  V  E  Y  A  S  D  S  I  I  I  K  E  G  D  V  G  S  I  V  Y  V  M
                                        cGMP-binding domain 1
     E  E  G  R  V  E  V  S  R  E  N  K  Y  L  S  T  M  T  S  G  K  V  F  G  E  L
V1 TGGAAGAGGGACGAGTGGAGGTGAGCAGAGAAAATAAATATCTTAGTACTATGACCTCCGGAAAAGTGTTCGGAGAGTTG 1200
V2 TGGAAGAGGGACGAGTGGAGGTGAGCAGAGAAAATAAATATCTTAGTACTATGACCTCCGGAAAAGTGTTCGGAGAGTTG 1118
     E  E  G  R  V  E  V  S  R  E  N  K  Y  L  S  T  M  T  S  G  K  V  F  G  E  L

    A  I  L  Y  N  C  K  R  T  A  T  I  K  A  A  T  D  C  K  L  W  A  I  E  R  Q  C
V1 GCTATTTTATACAACTGCAAGCGTACTGCCACTATTAAAGCTGCTACGGACTGTAAGCTATGGGCGATCGAACGACAATG 1280
V2 GCTATTTTATACAACTGCAAGCGTACTGCCACTATTAAAGCTGCTACGGACTGTAAGCTATGGGCGATCGAACGACAATG 1198
    A  I  L  Y  N  C  K  R  T  A  T  I  K  A  A  T  D  C  K  L  W  A  I  E  R  Q  C

     F  Q  T  I  M  M  R  T  G  L  I  R  Q  T  E  Y  T  D  F  L  K  S  V  P  I  F  K
V1 CTTCCAAACAATTATGATGAGGACTGGTCTCATAAGGCAAACGGAATATACCGATTTCCTCAAGAGCGTTCCAATATTCA 1360
V2 CTTCCAAACAATTATGATGAGGACTGGTCTCATAAGGCAAACGGAATATACCGATTTCCTCAAGAGCGTTCCAATATTCA 1278
     F  Q  T  I  M  M  R  T  G  L  I  R  Q  T  E  Y  T  D  F  L  K  S  V  P  I  F  K

     D  L  P  E  E  T  L  I  K  I  S  D  V  L  E  E  T  F  Y  N  A  G  D  Y  I  I
V1 AAGACCTGCCAGAAGAAACACTTATAAAGATTTCGGACGTTTTGGAAGAGACGTTTTACAATGCTGGAGACTATATAATC 1440
V2 AAGACCTGCCAGAAGAAACACTTATAAAGATTTCGGACGTTTTGGAAGAGACGTTTTACAATGCTGGAGACTATATAATC 1358
     D  L  P  E  E  T  L  I  K  I  S  D  V  L  E  E  T  F  Y  N  A  G  D  Y  I  I
```

```
   R  Q  G  A  R  G  D  T  F  F  I  N  K  G  K  V  K  V  T  I  K  Q  S  N  N  A
V1 AGACAAGGTGCCCGGGGAGACACGTTCTTCATAATCAACAAAGGAAAGGTTAAAGTCACCATAAAACAATCGAACAACGC 1520
V2 AGACAAGGTGCCCGGGGAGACACGTTCTTCATAATCAACAAAGGAAAGGTTAAAGTCACCATAAAACAATCGAACAACGC 1438
   R  Q  G  A  R  G  D  T  F  F  I  N  K  G  K  V  K  V  T  I  K  Q  S  N  N  A
                         cGMP-binding domain 2
    E  D  K  Y  I  R  T  L  Q  K  G  D  F  F  G  E  K  A  L  Q  G  D  D  L  R  T  A
V1 TGAAGACAAGTACATCAGAACCCTGCAAAAGGGTGACTTTTTCGGCGAAAAAGCGTTACAAGGCGATGACTTGCGTACCG 1600
V2 TGAAGACAAGTACATCAGAACCCTGCAAAAGGGTGACTTTTTCGGCGAAAAAGCGTTACAAGGCGATGACTTGCGTACCG 1518
    E  D  K  Y  I  R  T  L  Q  K  G  D  F  F  G  E  K  A  L  Q  G  D  D  L  R  T  A

     N  I  I  A  C  D  P  D  G  V  S  C  L  V  I  D  R  E  T  F  N  Q  L  I  A  G
V1 CGAACATAATCGCATGCGACCCCGACGGCGTATCGTGTTTGGTCATAGACCGGGAGACATTCAACCAACTGATTGCCGGA 1680
V2 CGAACATAATCGCATGCGACCCCGACGGCGTATCGTGTTTGGTCATAGACCGGGAGACATTCAACCAACTGATTGCCGGA 1598
     N  I  I  A  C  D  P  D  G  V  S  C  L  V  I  D  R  E  T  F  N  Q  L  I  A  G

   L  D  E  I  R  T  R  Y  K  D  D  D  V  L  G  R  M  S  S  T  N  K  E  F  Q  N  L
V1 CTAGACGAGATACGCACAAGATACAAAGACGACGATGTCTTGGGACGAATGAGCTCTACAAACAAGGAATTCCAAAACCT 1760
V2 CTAGACGAGATACGCACAAGATACAAAGACGACGATGTCTTGGGACGAATGAGCTCTACAAACAAGGAATTCCAAAACCT 1678
   L  D  E  I  R  T  R  Y  K  D  D  D  V  L  G  R  M  S  S  T  N  K  E  F  Q  N  L

    K  L  S  D  L  Q  V  L  A  T  L  G  V  G  G  F  G  R  V  E  L  V  Q  V  N  S  D
V1 AAAACTTTCCGACCTACAAGTCTTAGCCACTTTGGGCGTGGGTGGTTTCGGTCGTGTGGAACTCGTCCAAGTAAACAGCG 1840
V2 AAAACTTTCCGACCTACAAGTCTTAGCCACTTTGGGCGTGGGTGGTTTCGGTCGTGTGGAACTCGTCCAAGTAAACAGCG 1758
    K  L  S  D  L  Q  V  L  A  T  L  G  V  G  G  F  G  R  V  E  L  V  Q  V  N  S  D

     T  S  R  S  F  A  L  K  Q  M  K  K  S  Q  I  V  E  T  R  Q  Q  Q  H  I  M  S
V1 ACACATCTAGATCGTTTGCCCTAAAACAAATGAAAAAGAGTCAAATTGTTGAAACGAGACAACAACAGCACATCATGTCA 1920
V2 ACACATCTAGATCGTTTGCCCTAAAACAAATGAAAAAGAGTCAAATTGTTGAAACGAGACAACAACAGCACATCATGTCA 1838
     T  S  R  S  F  A  L  K  Q  M  K  K  S  Q  I  V  E  T  R  Q  Q  Q  H  I  M  S

   E  K  E  I  M  G  E  A  N  C  E  F  I  V  K  L  F  K  T  F  K  D  Q  K  Y  L  Y
V1 GAAAAAGAGATCATGGGAGAAGCCAACTGTGAATTCATTGTTAAGTTGTTCAAGACGTTCAAAGACCAAAAATACTTGTA 2000
V2 GAAAAAGAGATCATGGGAGAAGCCAACTGTGAATTCATTGTTAAGTTGTTCAAGACGTTCAAAGACCAAAAATACTTGTA 1918
   E  K  E  I  M  G  E  A  N  C  E  F  I  V  K  L  F  K  T  F  K  D  Q  K  Y  L  Y

    M  L  M  E  S  C  L  G  G  E  L  W  T  I  L  R  D  K  G  H  F  D  D  S  T  T  R
V1 TATGCTCATGGAGTCTTGTCTTGGTGGAGAATTGTGGACTATACTGAGAGACAAAGGACATTTTGATGATTCTACCACAC 2080
V2 TATGCTCATGGAGTCTTGTCTTGGTGGAGAATTGTGGACTATACTGAGAGACAAAGGACATTTTGATGATTCTACCACAC 1998
    M  L  M  E  S  C  L  G  G  E  L  W  T  I  L  R  D  K  G  H  F  D  D  S  T  T  R

     F  Y  T  G  C  V  V  E  A  F  D  Y  L  H  S  R  N  I  I  Y  R  D  L  K  P  E
V1 GTTTCTACACCGGATGTGTTGTTGAAGCATTCGACTACTTACATTCTCGCAACATAATTTATAGAGATCTGAAACCTGAA 2160
V2 GTTTCTACACCGGATGTGTTGTTGAAGCATTCGACTACTTACATTCTCGCAACATAATTTATAGAGATCTGAAACCTGAA 2078
     F  Y  T  G  C  V  V  E  A  F  D  Y  L  H  S  R  N  I  I  Y  R  D  L  K  P  E
                              cGMP-kinase domain
   N  L  L  L  D  I  T  G  Y  V  K  L  V  D  F  G  F  A  K  K  L  H  N  G  R  K  T
V1 AATCTACTTTTAGATATCACTGGTTATGTGAAATTGGTCGATTTTGGTTTTGCTAAAAAACTACATAACGGAAGAAAAAC 2240
V2 AATCTACTTTTAGATATCACTGGTTATGTGAAATTGGTCGATTTTGGTTTTGCTAAAAAACTACATAACGGAAGAAAAAC 2158
   N  L  L  L  D  I  T  G  Y  V  K  L  V  D  F  G  F  A  K  K  L  H  N  G  R  K  T

    W  T  F  C  G  T  P  E  Y  V  A  P  E  V  I  L  N  R  G  H  D  I  S  A  D  Y  W
V1 CTGGACATTCTGTGGAACACCAGAATATGTTGCCCCTGAAGTGATTCTCAACAGAGGTCATGATATTAGTGCTGATTACT 2320
V2 CTGGACATTCTGTGGAACACCAGAATATGTTGCCCCTGAAGTGATTCTCAACAGAGGTCATGATATTAGTGCTGATTACT 2238
    W  T  F  C  G  T  P  E  Y  V  A  P  E  V  I  L  N  R  G  H  D  I  S  A  D  Y  W

     S  L  G  V  L  M  F  E  L  L  T  G  T  P  P  F  T  G  A  D  P  M  K  T  Y  N
V1 GGTCCTTGGGAGTACTTATGTTTGAACTACTTACCGGTACACCACCATTCACTGGAGCAGATCCTATGAAAACTTATAAC 2400
V2 GGTCCTTGGGAGTACTTATGTTTGAACTACTTACCGGTACACCACCATTCACTGGAGCAGATCCTATGAAAACTTATAAC 2318
     S  L  G  V  L  M  F  E  L  L  T  G  T  P  P  F  T  G  A  D  P  M  K  T  Y  N

   I  I  L  K  G  I  D  A  I  E  F  P  R  N  I  T  R  N  A  R  V  L  I  K  K  L  C
V1 ATAATTCTAAAAGGCATTGATGCAATAGAATTTCCAAGAAATATAACAAGAAATGCTAGAGTTTTAATTAAGAAATTATG 2480
V2 ATAATTCTAAAAGGCATTGATGCAATAGAATTTCCAAGAAATATAACAAGAAATGCTAGAGTTTTAATTAAGAAATTATG 2398
   I  I  L  K  G  I  D  A  I  E  F  P  R  N  I  T  R  N  A  R  V  L  I  K  K  L  C

    R  D  N  P  A  E  R  L  T  E  V  Q  K  H  K  W  F  D  G  F  N  W  E  G  L  R  N
V1 CAGAGATAATCCTGCTGAACGGCTAACTGAAGTACAAAAACACAAGTGGTTTGATGGTTTCAATTGGGAAGGACTACGTA 2560
V2 CAGAGATAATCCTGCTGAACGGCTAACTGAAGTACAAAAACACAAGTGGTTTGATGGTTTCAATTGGGAAGGACTACGTA 2478
    R  D  N  P  A  E  R  L  T  E  V  Q  K  H  K  W  F  D  G  F  N  W  E  G  L  R  N

     R  T  L  T  P  P  I  L  P  K  V  R  S  A  I  D  T  S  N  F  D  N  Y  P  P  D
V1 ATCGAACCCTAACTCCTCCTATCTTGCCAAAGGTGCGAAGTGCTATAGATACAAGCAATTTTGACAATTATCCGCCTGAC 2640
V2 ATCGAACCCTAACTCCTCCTATCTTGCCAAAGGTGCGAAGTGCTATAGATACAAGCAATTTTGACAATTATCCGCCTGAC 2558
     R  T  L  T  P  P  I  L  P  K  V  R  S  A  I  D  T  S  N  F  D  N  Y

   A  D  S  P  P  P  D  D  N  S  G  W  D  V  N  F
V1 GCAGACAGCCCACCACCAGATGATAATTCAGGATGGGATGTAAATTTCTAATATGTAAAAAACAATGATTATTGTTTGTC 2720
V2 GCAGACAGCCCACCACCAGATGATAATTCAGGATGGGATGTAAATTTCTAATATGTAAAAAACAATGATTATTGTTTGTC 2638
   A  D  S  P  P  P  D  D  N  S  G  W  D  V  N  F
```

```
V1  CGCCTCAACGATTTTACTCTATGGTTTCAAATTACCCATGTTTTACATTCTAGTTATGTCTTACAAATTAATTTAGTCTT  2800
V2  CGCCTCAACGATTTTACTCTATGGTTTCAAATTACCCATGTTTTACATTCTAGTTATGTCTTACAAATTAATTTAGTCTT  2718

V1  GTTAATTATTCAGTGTACTACCATATGAAAATAATAAGTGTTAGATTATTTGATATTATATTTCAATTTTTGTTAACCAT  2880
V2  GTTAATTATTCAGTGTACTACCATATGAAAATAATAAGTGTTAGATTATTTGATATTATATTTCAATTTTTGTTAACCAT  2798

V1  ACCATTTAAATTTTGTTTTTATATAAATAAAATATTTGTTATACCCCTCCCCCCACCCTCTGGTAGATATCCATAATTAT  2960
V2  ACCATTTAAATTTTGTTTTTATATAAATAAAATATTTGTTATACCCCTCCCCCCACCCTCTGGTAGATATCCATAATTAT  2878

V1  CTACATTTACGAATTTAATATACAATAAGTGCCTTTCAATTTTCTGAACACATATTATGGCAGAATATACAACTGTCATT  3040
V2  CTACATTTACGAATTTAATATACAATAAGTGCCTTTCAATTTTCTGAACACATATTATGGCAGAATATACAACTGTCATT  2958

V1  TATTAATTTATTATAGTTAAGATTGCGCGAGTTTTATTTTTTTCCAATTAAGTAAAAGTGTATGTTGGTTATTACTTTTC  3120
V2  TATTAATTTATTATAGTTAAGATTGCGCGAGTTTTATTTTTTTCCAATTAAGTAAAAGTGTATGTTGGTTATTACTTTTC  3038

V1  TTCTTGTATAGTTTGATTATTGATTTATGTGTTTTAGTATTTATAAATAATTTTTATATGGAAAAGTGTCCAGATATAAA  3200
V2  TTCTTGTATAGTTTGATTATTGATTTATGTGTTTTAGTATTTATAAATAATTTTTATATGGAAAAGTGTCCAGATATAAA  3118

V1  AATTATACATTAAAATATTATGTTATAACATAGTAAGATCACAATAACTATGAATTATTTTTTATGATTGTTATTATTGT  3280
V2  AATTATACATTAAAATATTATGTTATAACATAGTAAGATCACAATAACTATGAATTATTTTTTATGATTGTTATTATTGT  3198

V1  TGCTCTTTGAATAGTCCTTATCCAGTGAGTCCCTTGACTTTATATATATATATATTATATATACACTTATATATTTTTTA  3360
V2  TGCTCTTTGAATAGTCCTTATCCAGTGAGTCCCTTGACTTTATATATATATATATTATATATACACTTATATATTTTTTA  3278

V1  ATTAATAAAAATTATTTTACACATTGGACAAAAAAAAAAAAAAAAAAAAAAAAAAAAAAAA                     3432
V2  ATTAATAAAAATTATTTTACACATTGGACAAAAAAAAAAAAAAAAAAAAAAAAAAAAAAAA                     3341
```

Figure S1
