# Supplementary figures and images for: Environment Exploration and Colonization Behavior of the Pea Aphid Associated with the Expression of the foraging Gene
Source: PLoS One. 2013 May 29;8(5):e65104. doi: 10.1371/journal.pone.0065104 (PMC3667181; doi:10.1371/journal.pone.0065104)

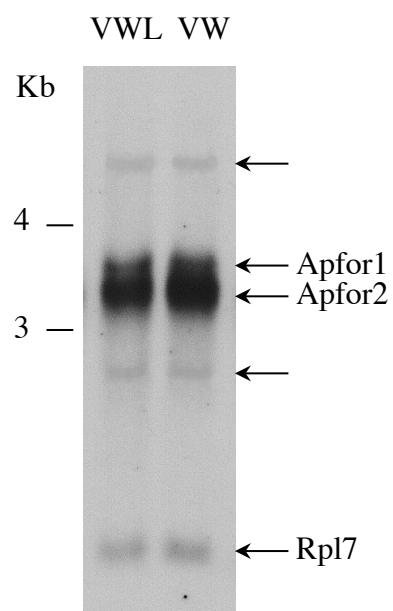

Figure S2

Supplement: Figure S2 — Northern blot analysis of the Apfor expression. 6 µg of polyA+ mRNAs from wingless adults were used. The 406 bp probe overlapping the two cGMP-binding domains of Apfor was digoxigenine-labelled using the PCR DIG probe synthesis kit from Roche Diagnostics (Germany). A RPL7 fragment was used as control. (PDF) [file pone.0065104.s002.pdf]
