# Supplementary material for: Environment Exploration and Colonization Behavior of the Pea Aphid Associated with the Expression of the foraging Gene
Source: PLoS One. 2013 May 29;8(5):e65104. doi: 10.1371/journal.pone.0065104 (PMC3667181; doi:10.1371/journal.pone.0065104)
Supplement: Table S1 — Oligonucleotide primers used for quantitative real-time PCR. (PDF) [file pone.0065104.s003.pdf]

Table S1. Oligonucleotide primers used for quantitative real-time PCR

Gene	Forward primer	Reverse primer
Apfor kinase domain	TGCTGAACGGCTAACTGAAGTA	ACTTCGCACCTTTGGCAAGATA
Apfor1	ACCCACTAGTACGCTGCATCA	ACCATCCAGAAGCGACGTAAGA
Apfor2	CGAAACCACACTGCAGCTACAT	GGTCGCCACAAACTATGCGAT
Rpl7	ACTGTTCAGATTGCGTCAGATC	AGTTCCCTTACGCTCTTCAAGT
